# Microbial cycling of isoprene, the most abundantly produced biological volatile organic compound on Earth

**DOI:** 10.1038/s41396-018-0072-6

**Published:** 2018-02-20

**Authors:** Terry J. McGenity, Andrew T. Crombie, J. Colin Murrell

**Affiliations:** 1 School of Biological Sciences, University of Essex, Wivenhoe Park, Colchester, CO4 3SQ UK; 2 School of Biological Sciences, University of East Anglia, Norwich Research Park, Norwich, NR4 7TJ UK; 3 School of Environmental Sciences, University of East Anglia, Norwich Research Park, Norwich, NR4 7TJ UK

**Keywords:** Biogeochemistry, Water microbiology, Soil microbiology

## Abstract

Isoprene (2-methyl-1,3-butadiene), the most abundantly produced biogenic volatile organic compound (BVOC) on Earth, is highly reactive and can have diverse and often detrimental atmospheric effects, which impact on climate and health. Most isoprene is produced by terrestrial plants, but (micro)algal production is important in aquatic environments, and the relative bacterial contribution remains unknown. Soils are a sink for isoprene, and bacteria that can use isoprene as a carbon and energy source have been cultivated and also identified using cultivation-independent methods from soils, leaves and coastal/marine environments. Bacteria belonging to the Actinobacteria are most frequently isolated and identified, and Proteobacteria have also been shown to degrade isoprene. In the freshwater-sediment isolate, *Rhodococcus* strain AD45, initial oxidation of isoprene to 1,2-epoxy-isoprene is catalyzed by a multicomponent isoprene monooxygenase encoded by the genes *isoABCDEF*. The resultant epoxide is converted to a glutathione conjugate by a glutathione *S*-transferase encoded by *isoI*, and further degraded by enzymes encoded by *isoGHJ*. Genome sequence analysis of actinobacterial isolates belonging to the genera *Rhodococcus, Mycobacterium* and *Gordonia* has revealed that *isoABCDEF* and *isoGHIJ* are linked in an operon, either on a plasmid or the chromosome. In *Rhodococcus* strain AD45 both isoprene and epoxy-isoprene induce a high level of transcription of 22 contiguous genes, including *isoABCDEF* and *isoGHIJ*. Sequence analysis of the *isoA* gene, encoding the large subunit of the oxygenase component of isoprene monooxygenase, from isolates has facilitated the development of PCR primers that are proving valuable in investigating the ecology of uncultivated isoprene-degrading bacteria.

## Background and isoprene production

### Isoprene’s properties, abundance, and climate impact

Isoprene (2-methyl-1,3-butadiene; CH_2_=C(CH_3_)CH=CH_2_) is an abundant BVOC, with atmospheric emissions of around 500 Tg C year^−1^, which is approximately equal to that of methane and also of the same magnitude as emissions of all other BVOCs [1]. Isoprene’s abundance, volatility (boiling point of 34 °C), and high reactivity (due to carbon–carbon double bonds) result in it having a major impact on climate [2], the precise mechanisms and outcomes of which are varied and complex. Key effects are summarized in the following text. (1) Isoprene’s reactivity, e.g., with hydroxyl radicals, reduces the oxidizing capacity in the atmosphere, leading to a prolonged lifetime for the greenhouse gas, methane, thus exacerbating global warming [3]. (2) Nitric oxide (NO), when present at high concentrations, reacts with isoprene to produce nitrogen dioxide (NO_2_) that, via photolysis, increases levels of tropospheric ozone [2], which is a greenhouse gas and detrimental to plant and animal health [4]. The precise reactions and effects depend on environmental conditions, such as light intensity and nitrogen oxide concentrations [2]. (3) Atmospheric oxidation of isoprene forms secondary organic aerosols [5], with potentially negative implications for air quality and health [6]. Secondary organic aerosols also absorb and scatter solar radiation and encourage cloud formation, resulting in global cooling. However, the extent to which isoprene helps or even inhibits formation of secondary organic aerosols is debated and context-dependent [84].

### Isoprene’s production, biosynthetic pathways, biological functions, and biotechnological potential

**Fig. 1:**
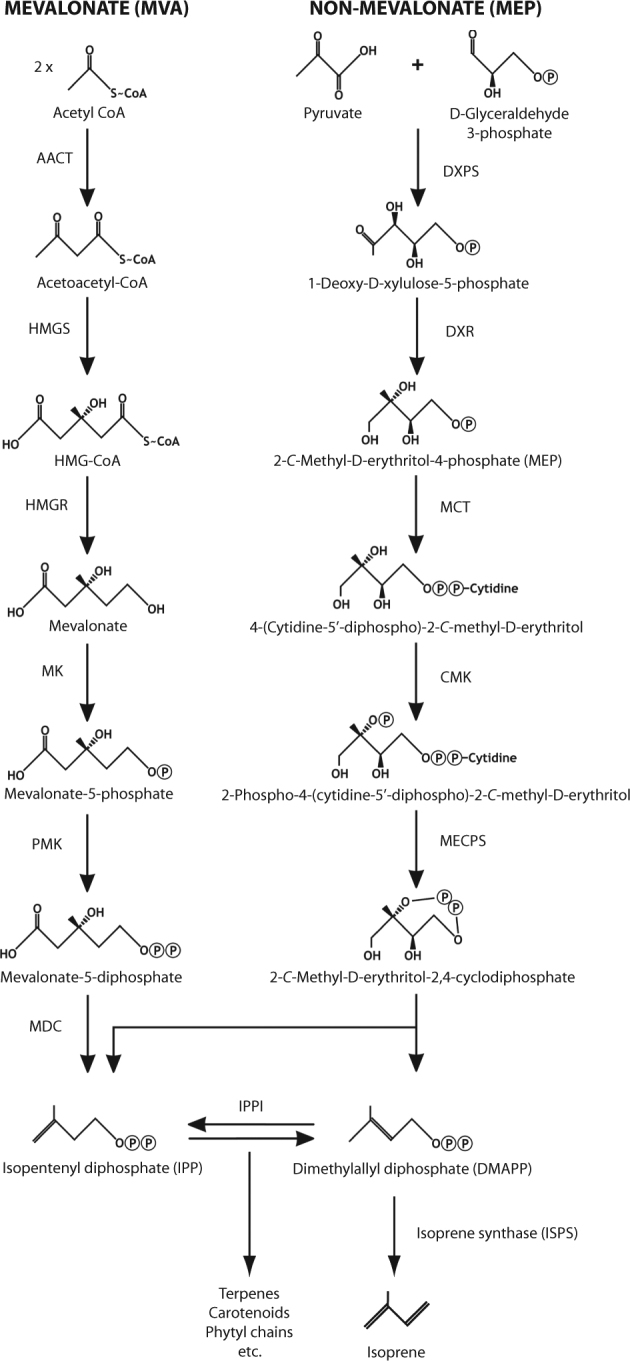
Biosynthesis of isoprene and isoprenoids via the mevalonate (MVA) and non-mevalonate or methylerythritol phosphate (MEP) pathways, with isopentenyl diphosphate (IPP) and dimethylallyl diphosphate (DMAPP) as key intermediates. Mevalonate pathway enzymes shown are: AACT acetoacetyl-CoA thiolase, HMGS 3-hydroxy-3-methylglutaryl-CoA synthase, HMGR 3-hydroxy-3-methylglutaryl-CoA reductase, MK mevalonate kinase, PMK phosphomevalonate kinase, MDC mevalonate 5-diphosphate decarboxylase. Note that an alternative route exists in Archaea for the conversion of mevalonate-5-phosphate to IPP [81]. MEP Pathway enzymes shown are: DXPS 1-deoxyxylulose 5-phosphate synthase, DXR 1-deoxyxylulose 5-phosphate reductoisomerase, MCT 2-*C*-methyl-D-erythritol 4-phosphate cytidyl transferase, CMK 4-(cytidine 5′-diphospho)-2-*C*-methyl-D-erythritol kinase, MECPS 2-*C*-methyl-D-erythritol-2,4-cyclodiphosphate synthase. The intermediate, (E)-4-hydroxy-3-methylbut-2-en-1-yl diphosphate (HMBPP), from 2-*C*-methyl-d-erythritol-2,4-cyclodiphosphate to IPP and DMAPP, is not shown. Enzymes common to both pathways are: IPPI isopentenyl diphosphate isomerase, ISPS isoprene synthase. Modified from Lange et al. [82] and Steinke et al. [83]

Isoprenoids (or terpenes/terpenoids, consisting of two or more isoprene units) are a large and diverse class of molecules produced by all free-living organisms. They include or form part of the following biomolecules: hopanoids, sterols, archaeal lipids, carotenoids, chlorophylls and quinones, various hormones and signaling molecules [7]. Isoprenoids are synthesized by condensations of isopentenyl diphosphate (IPP) and its isomer dimethylallyl diphosphate (DMAPP; Fig. 1). Two pathways lead to the biosynthesis of these key intermediates—the mevalonate (MVA) pathway and the methylerythritol phosphate (MEP) pathway, which is also referred to as the non-mevalonate or D(O)XP pathway [7]. The MVA pathway is found in animals, fungi, Archaea, some Bacteria and in the cytosol of plants, whereas the MEP pathway is found in chloroplasts and most Bacteria [7]. The picture in eukaryotic protists/algae is less straightforward, and could provide an insight into their complex evolution, specifically their plastid origins and replacements [7, 8].

**Fig. 2:**
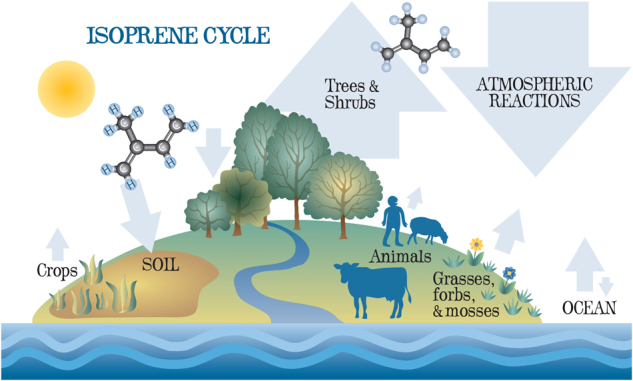
The isoprene cycle. Arrow thickness provides a schematic illustration of the relative contribution, where known, of different sources (up arrow) and sinks (down arrow) of isoprene. [A forb is a vascular plant without significant above-ground woody tissue that is not a grass, sedge or rush]

Isoprene is produced by some Bacteria, fungi, protists/algae, and animals [9–12]. The vast majority of isoprene, however, is produced by terrestrial plants  [1, 13], Fig. 2. To our knowledge, archaeal production of isoprene has not been tested, but many Bacteria produce isoprene, at least in those terrestrial strains from a relatively limited number of phyla tested: Proteobacteria, Actinobacteria and Firmicutes [10, 11, 14, 15]. *Bacillus* and relatives have been found to be the most prolific producers of isoprene, for both terrestrial [11] and marine (G. Murphy and T.J. McGenity, unpub.) species. In *Bacillus subtilis*, isoprene was produced during three distinct phases of growth, but it remains to be confirmed whether isoprene is an overflow metabolite or serves as a signaling molecule involved in cell/spore development [10, 16, 17]. Isoprene may also play a role in interspecies signaling [10]; for example it has been shown to repel microbe-grazing Collembola [18]. Isoprene production was also enhanced when *Bacillus subtilis* was subjected to hydrogen peroxide [19, 20] and supra-optimal temperature and salinity [20], hinting at a role in stress protection, as seen in plants.

Isoprene synthase (IspS), the Mg^2+^-requiring terpenoid synthase that catalyzes elimination of pyrophosphate from DMAPP to produce isoprene (Fig. 1), has been structurally characterized in the tree species Populus × canescens [21], and the underlying genetics determined in several plant species. There is no evidence that the bacterial isoprene synthase is homologous to that from plants. Extensive searches of plant isoprene synthase sequences and relevant motifs revealed no homologs in the bacterial databases (Odubo, McGenity and Hough, unpublished). Isoprene synthase was partially purified from *Bacillus subtilis*, but proved to be labile, thus allowing only initial investigation, e.g., demonstrating a lower divalent cation requirement and lower pH optimum than isoprene synthases from the chloroplasts of plants [16]. An alternative route to isoprene production was proposed by Ge et al. [22], who found that IspH, the final enzyme of the MEP pathway, converts HMBPP to isoprene, thus bypassing DMAPP [HMBPP or (E)-4-hydroxy-3-methylbut-2-en-1-yl diphosphate is an intermediate metabolite in the transformation of 2-*C*-methyl-d-erythritol-2,4-cyclodiphosphate to IPP and DMAPP]. However, IspH gene expression was not correlated with higher isoprene production in the transcriptome-based study of [19]. Therefore, identification of the precise mechanism(s) of isoprene synthesis in Bacteria and the responsible enzyme(s) remains an area of active research.

Isoprene is an important commodity chemical, currently produced from petroleum, with the potential to serve as an aviation fuel and a feedstock for numerous products, including its polymer, rubber [23, 24]. Therefore, there have been many papers and patents reporting its recombinant biosynthesis in microbes by heterologous expression of codon-optimized isoprene synthase genes from plants, due to the lack of genetic information about microbial isoprene synthases. This strategy has been coupled with methods to enhance carbon flux to DMAPP [24]. To date, the highest titers have come from recombinant expression in *Escherichia coli* [23] rather than in *Saccharomyces cerevisiae* or the cyanobacterium, *Synechocystis* sp. [24]. Ye et al. [24] summarize these strategies, as well as other approaches, such as recombinant isoprene production with methanol as a feedstock, that are emerging in the patent literature.

Terrestrial plants produce more than 90% of isoprene globally, with the biggest contribution from trees and shrubs, especially in the tropics [13]. There can be a lot of variation in isoprene production within the same genus (e.g., European oaks), indicating that isoprene’s function can be replaced by other metabolites [13]. Given that ~2% of carbon fixation goes towards synthesis of isoprene in most isoprene-producing plants, and that 20 molecules of ATP and 14 molecules of NADPH are required for the biosynthesis of each isoprene molecule [25], it is not surprising that isoprene, which cannot be stored in the leaves, has important biological roles. Two key functions have been shown: (1) rapid alleviation of thermal stress, particularly in response to heat flecks [13]; (2) protection from reactive oxygen (and nitrogen) species (ROS) formed by a range of abiotic stresses [26]. The degree to which intercalation of isoprene into the membrane and the ROS-quenching effect of its double carbon–carbon bonds, respectively, are responsible for these effects, remains a topic of debate [27, 28]. Numerous other roles have been shown for isoprene, including signaling, e.g., influencing insect herbivory [29] and regulating plant growth [30].

## Ecology, physiology, and biogeochemistry of the isoprene cycle

### Terrestrial environments

It has been known for 20 years that microbes in soil can act as a biological sink for isoprene. In laboratory experiments with temperate, tropical and boreal forest soils, isoprene, added at 385 ppbv, was generally rapidly depleted, while in field chambers placed in a temperate forest, isoprene, added at 508 ppbv, was consumed to below the 5 ppbv detection limit within an hour [31, 32]. In-situ biological consumption of isoprene was also demonstrated in temperate agriforest (*Populus deltoides*) mesocosms and model tropical rain-forest mesocosms [33, 34]. More recently, in continuous-flow experiments conducted by Gray et al. [35], temperate forest soils consumed isoprene over a range of concentrations (2–200 ppbv) with a rate of 62 pmol g^−1^ h^−1^ at 20 ppbv. These experiments confirmed the potential for soil microbes to consume isoprene released locally in soils as well as from the atmosphere. Bacteria tentatively assigned to the genera *Rhodococcus*, *Nocardia, Arthrobacter* (Actinobacteria) and *Alcaligenes* (Proteobacteria) were isolated from isoprene enrichments with soil and grew on isoprene as sole carbon and energy source [31, 36–38]. Rubber-contaminated soil has also yielded aerobic isoprene-degrading *Pseudomonas*, *Klebsiella* and *Alcaligenes* strains [39]. However, with the exception of *Rhodococcus* strain AD45 [see later section] isolated from freshwater sediment [40, 41], these terrestrial strains were not characterized in detail.

Recently a number of isoprene-degrading *Rhodococcus* species from soils and leaves of isoprene-producing trees such as Poplar and Willow have been isolated and are currently being characterized ([42]; Murrell, McGenity, Crombie, Murphy et al. unpublished), suggesting that these metabolically versatile Bacteria may be major players in the biological isoprene cycle. To our knowledge, no anaerobes, Archaea or fungi that grow on isoprene as carbon source have been isolated.

Isoprene-degrading Bacteria co-metabolically oxidize the common pollutant trichloroethene (TCE) and other chlorinated ethenes [36, 40], owing to the low-specificity of isoprene monooxygenase (the epoxide-forming soluble di-iron center monooxygenase discussed in detail later). Given the abundance of isoprene, this process may be an important route for removal of chlorinated ethenes from oxic environments. Conversely, there is evidence of co-oxidation of isoprene by (poly)aromatic dioxygenases from *Pseudomonas putida*, in which the first intermediates are diols rather than epoxides [43]. It is also probable that isoprene is co-metabolized by the monooxygenases of alkene-oxidizing bacteria, resulting in a toxic epoxide that may not be further metabolized, and would thus kill cells. The relative contribution of co-metabolic degradation of isoprene, compared with direct oxidation by microbes that use isoprene as a source of carbon and energy, remains to be elucidated.

### Marine environments

Marine isoprene emissions, primarily from photosynthetic microalgae, generally correlate well with photosynthetic activity (using chlorophyll *a* concentrations as a proxy) in depth profiles, natural and fertilizer-induced algal blooms and diel cycles [44–46]. In contrast to the terrestrial environment, global marine isoprene-emission estimates vary about hundred-fold. A detailed review [46] and recent paper [47] provide a thorough discussion of the reasons behind this variation, but a synopsis is provided here. Bottom-up estimates (coupling of laboratory measurements of isoprene production by microalgae and satellite measurements of phytoplankton biomass (chlorophyll *a*)) give estimates of 0.1–1 Tg C year^−1^ [46, 48–50]. Top-down methods (coupling of measurements of isoprene flux at sea and global inverse atmospheric modeling) give global emission estimates of 1.7–11.6 Tg C y^−1^ [46, 48, 49]. Although marine emissions are low compared with those from land, they can have a disproportionately large influence on aerosol formation and the atmosphere’s oxidation capacity in remote and pristine regions [48, 51, 52]. This large variation in estimates of global isoprene emissions from marine environments may be explained by a number of factors that are not always sufficiently accounted for in models, including the mainly biological factors outlined here: (1) photochemically-derived isoprene, e.g., from breakdown of fatty acids in the sea-surface microlayer, which is not considered in bottom-up methods [53]; (2) limited data, including from highly productive coastal zones and different oceans and accounting for seasonal variation (e.g., [44, 47, 54, 55]); (3) differences in isoprene production under variable and stressful in-situ conditions compared with laboratory cultures [9, 52]; (4) phylogenetic and latitudinal variation in isoprene production by microalgal species [9, 48, 56]. Points 3 and 4 are most dramatically exemplified by Srikanta Dani et al. [57], who suggest that diatoms alone emit 4.8 Tg C y^−1^ of isoprene, which greatly exceeds current bottom-up estimates for all taxa in marine ecosystems. It has recently been found that freshwater lakes, via their resident microalgae, emit isoprene to the atmosphere [58].

The functions of isoprene production in marine microalgae are not well understood. Water buffers aquatic organisms from rapid changes in temperature, which may in part explain lower isoprene production compared with many terrestrial species. However, as with plants, higher temperature and light intensity result in more isoprene production [9, 44, 47, 51, 52]. In an extensive ocean survey, Hackenberg et al. [47] found a consistent correlation between isoprene production and the sum of photo-protective carotenoids. Srikanta Dani and Loreto [59] argue that isoprene acts primarily as an antioxidant, protecting against reactive oxygen species that may be produced by diverse stressors, including light stress. Moreover, they extend this idea by suggesting that dimethylsulfide (DMS) serves the same purpose, and that different microalgal taxa preferentially emit DMS or isoprene to fulfill this role. The global implication of this notion is that more isoprene will be produced in the future due to climate-change-induced rise in sea-surface temperature favoring isoprene-emitting microalgae [59].

Another poorly understood aspect of the marine isoprene cycle is the role of microbial degradation. Ocean depth profiles of isoprene concentrations suggest that it is biologically consumed [45]. Therefore, Palmer and Shaw [50] incorporated a term for the rate of isoprene biodegradation in their bottom-up approach to model global isoprene emissions. However, this rate term was based on data from methyl bromide biodegradation, which was considered to be a functionally equivalent BVOC. Booge et al. [56] reported that the application of a lower value for isoprene biodegradation resulted in closer correspondence between observed and simulated marine isoprene flux. The justification for doing so was based on their currently unpublished biodegradation measurements using deuterated isoprene, which gave lower values than the rate used by Palmer and Shaw [50]. These data deficiencies clearly indicate that there is a need to obtain measurements of in-situ biodegradation of isoprene in marine environments.

Acuña Alvarez et al. [60] first demonstrated the presence of isoprene-degrading microbes along an estuary (Colne estuary, UK), in a brackish lagoon (Étang de Berre, France) and in marine waters (between the islands of Hoga and Kaledupa, Indonesia). Importantly, they showed that the isoprene-degrading bacteria consumed isoprene from the headspace of microalgal cultures, i.e., at environmentally relevant concentrations. Biodegradation was also found to be faster at lower isoprene concentrations. As in terrestrial environments, the most abundant marine microbes belonged to the Actinobacteria, specifically the genera *Rhodococcus* and *Mycobacterium*. But Indonesian seawater enrichments were co-dominated by the alphaproteobacterial genus *Stappia*. An isoprene-degrading strain belonging to this genus has subsequently been isolated from station L4 in the English Channel [61]. Cultivated isoprene degraders were nutritionally versatile, and nearly all of them were able to use short-chain *n-*alkanes as a source of carbon and energy [60]. Johnston et al. [61], investigating the Colne estuary and other UK marine waters, expanded our knowledge of the diversity of marine and coastal isoprene degraders as discussed in the next section.

## Physiology, biochemistry, genetics, genomics, and molecular ecology of isoprene degraders

**Fig. 3:**
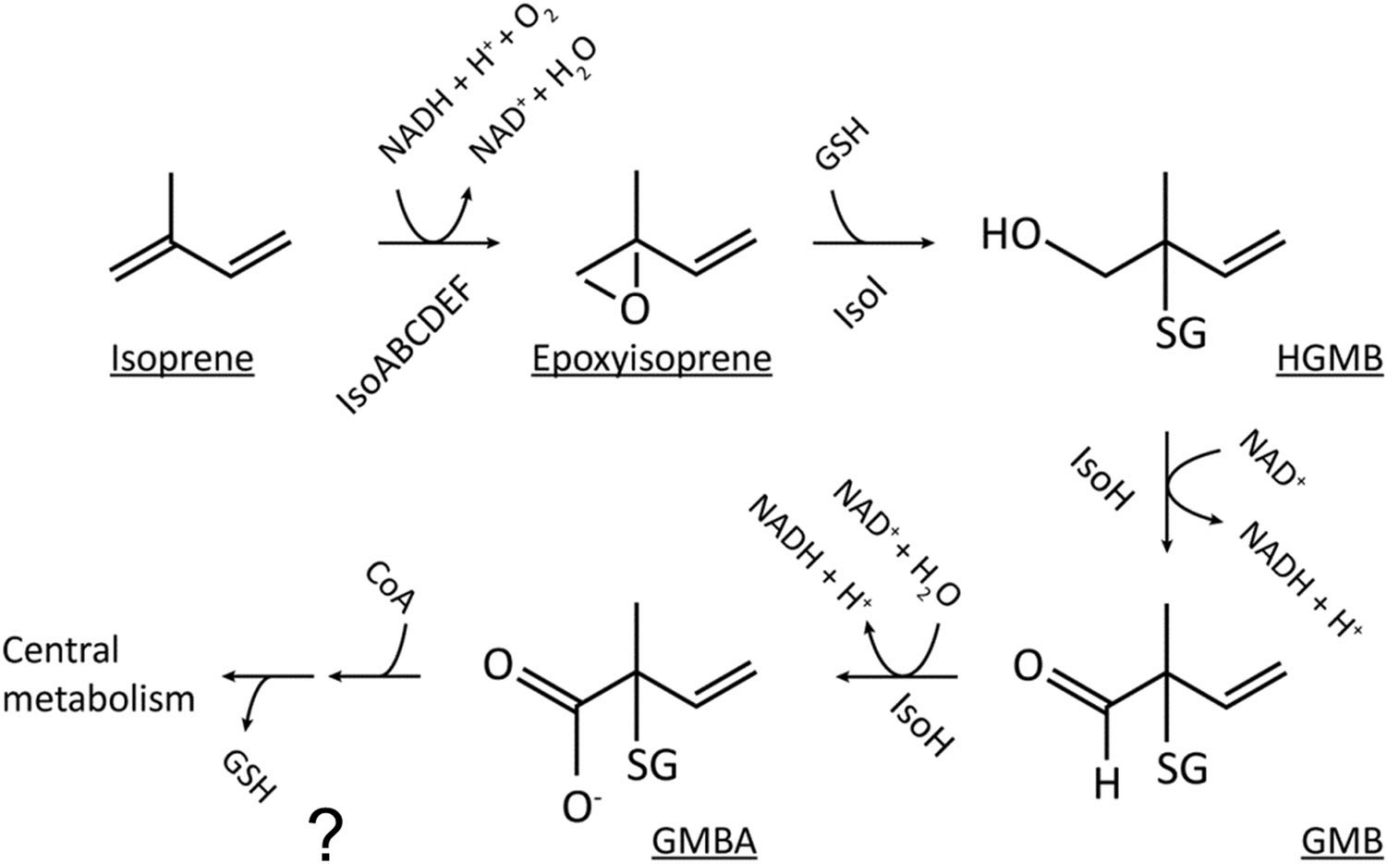
The pathway of isoprene degradation. HGMB 1-hydroxy-2-glutathionyl-2-methyl-3-butene, GMB 2-glutathionyl-2-methyl-3-butenal, GMBA 2-glutathionyl-2-methyl-3-butenoate, SG glutathione, GSH reduced glutathione [64]. The question mark indicates uncertainty in the details of the catabolic pathway from GMBA

Until recently, the only research on isoprene metabolism by bacteria was with the freshwater-sediment isolate *Rhodococcus* strain AD45 carried out by Janssen and colleagues. The initial oxidation of isoprene to 1,2-epoxy-isoprene is catalyzed by a multicomponent isoprene monooxygenase encoded by the genes *isoABCDEF* [41]. Although isoprene monooxygenase has not been purified, it is clear from gene sequence analysis that this enzyme is a soluble di-iron center monooxygenase (SDIMO) related to soluble methane monooxygenase, alkene monooxygenase, toluene monooxygenase and other enzymes of the SDIMO family [62, 63]. The reactive epoxide formed during the initial oxidation of isoprene (Fig. 3) is converted to a glutathione conjugate 1-hydroxy-2-glutathionyl-2-methyl-3-butene (HGMB) by a glutathione *S*-transferase (IsoI) and then by a dehydrogenase (IsoH) to 2-glutathionyl-2-methyl-3-butenoate (GMBA) [64]. The strategy of conjugating the epoxide with glutathione used by *Rhodococcus* strain AD45 is different from that of other alkene degraders, which usually overcome the toxicity of the epoxides by hydrolysis or formation of coenzyme M conjugates [65–67]. Apart from the first few steps in isoprene metabolism, the pathway in *Rhodococcus* strain AD45 has not been characterized and the further fate of GMBA is uncertain. It is assumed that subsequent removal of glutathione and beta-oxidation of these isoprene oxidation products enables *Rhodococcus* strain AD45 to grow on isoprene as a carbon source but subsequent steps in the metabolism of isoprene remain to be elucidated.

**Fig. 4:**
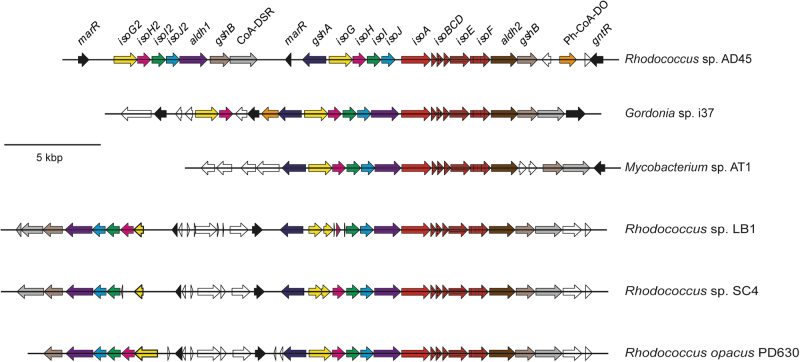
The genomic region surrounding the isoprene monooxygenase genes (*isoABCDEF*) of sequenced isoprene-degrading marine strains *Gordonia* sp. i37 and *Mycobacterium* sp. AT1 [61], terrestrial strains *Rhodococcus* sp. LB1 and *Rhodococcus* sp. SC4 [42] and terrestrial strain *Rhodococcus opacus* PD630 [69], in comparison to *Rhodococcus* sp. AD45 [68]. The isoprene monooxygenase genes are shown in red and other genes, homologous between the strains, are in matching colors. Genes encoding hypothetical proteins of unknown function and those without homologs in this region of the *Rhodococcus* sp. AD45 genome are in white. Note that the two copies of *isoG* and *isoH* in *Rhodococcus* sp. LB1 and *Rhodococcus* sp. SC4 could not be unambiguously assembled, resulting in multiple contigs for these strains that are shown aligned to the *Rhodococcus opacus* PD630 sequence, as reference. CoA-DSR coenzyme A disulfide reductase, Ph-CoA-DO phytanoyl coenzyme A dioxygenase

Sequencing of the genome of *Rhodococcus* strain AD45 (6.80 Mbp) has shown that all isoprene metabolic genes, *isoA* to *isoJ* (Fig. 4) are located on a 300 kbp megaplasmid. The genes *isoA*, *isoB*, and *isoE* encode a di-iron (αβγ)_2_ oxygenase component, *isoF* encodes a flavoprotein NADH reductase, *isoC* encodes a Rieske-type ferredoxin, and *isoD* encodes a coupling protein, which together form isoprene monooxygenase. Adjacent genes *isoGHIJ* encode enzymes in the subsequent steps in isoprene degradation. The *iso* genes are essential for isoprene metabolism since deletion of *isoA* by mutagenesis or removal of the plasmid by “curing” eliminates growth on isoprene [68]. A key feature of those isoprene-degrading bacteria for which genome sequences are available is the clustering of isoprene monooxygenase genes *isoABCDEF* with *isoGHIJ* (Fig. 4), genes that code for a putative coenzyme A transferase, a dehydrogenase and two glutathione transferases described previously [40, 41, 64]. *Rhodococcus* strain AD45 contains two copies of the *isoGHIJ* gene cluster, together with genes encoding glutathione biosynthesis. Identification of the genes required for isoprene metabolism has enabled the screening of genomes from other *Rhodococcus* species to search for extant but untested isoprene degraders. For example, the genome of *Rhodococcus opacus* PD630 [69], a well-studied and metabolically-versatile actinobacterium, not previously investigated for alkene metabolism, contains a similar *iso* gene cluster, and when tested, grew on isoprene [68]. A similar pattern is emerging for the clustering of *iso* genes in other isoprene degraders. For example, *Rhodococcus* strains from soil and leaves that grow on isoprene also contain *isoABCDEF* adjacent to *isoGHIJ* and other genes essential for isoprene metabolism [42], and marine isoprene-degrading *Gordonia* and *Mycobacterium* strains have similarly arranged genes ([61]; Fig. 4).

In the most well-characterized isoprene degrader, *Rhodococcus* strain AD45, expression of isoprene metabolic genes is inducible by isoprene but repressed by other carbon sources such as succinate or glucose [68]. Isoprene monooxygenase polypeptides are also readily observed in cell-free extracts of isoprene-grown cells. RNAseq experiments investigating transcriptional regulation of *iso* genes in strain AD45 revealed that both isoprene and epoxyisoprene induced a high level of transcription of 22 contiguous genes (Fig. 4), including *isoABCDEF* and *isoGHIJ*, which under these induced conditions represented over 25% of total transcripts observed [68]. Exact details of how isoprene monooxygenase gene expression is regulated, along with the nature of the promoters, transcriptional activators and inducers, are not known, but it is interesting to note that epoxyisoprene, the first oxidation product in the pathway is an inducer of expression of the *isoABCDEF* and *isoGHIJ* gene clusters [68]. It may be that the inducer is not isoprene itself but that a subsequent intermediate in isoprene oxidation is the key inducer for isoprene metabolism, as has been observed in the catabolism of aromatic compounds (reviewed in [70]). Most of these genes have a readily predicted function in isoprene metabolism, including isoprene monooxygenase, glutathione transferase, dehydrogenase, and glutathione biosynthesis genes. Other genes, annotated as encoding aldehyde dehydrogenases, disulfide reductase and three putative transcriptional regulators, are also found in this 22-gene cluster (Fig. 4), and are further targets to analyse isoprene metabolism and its regulation in *Rhodococcus* strain AD45 [68].

The identity and availability of genes encoding key enzymes of isoprene metabolism by bacteria have paved the way for cultivation-independent studies to determine the distribution, diversity and activity of isoprene degraders in the environment. The picture is emerging of isoprene metabolism being widespread across many genera of diverse bacteria [42, 60, 61, 71], and so development of 16S rRNA gene primers or probes to specifically detect isoprene degraders directly from the environment without coupling these with other techniques such as stable isotope probing (SIP) or Raman microspectroscopy (see later) will not be fruitful. A more promising approach is to design PCR primer sets targeting key genes involved in isoprene metabolism, an approach that has proven successful in targeting methane-oxidizing bacteria in the environment [72]. An obvious target is *isoA* encoding the large subunit of the oxygenase component of isoprene monooxygenase, an analogous gene to *mmoX* encoding the active site component of soluble methane monooxygenase, a related SDIMO enzyme. Alignment of *isoA* homologs from isoprene-degrading bacteria has enabled the design of PCR primers that tested negative with SDIMO genes from non-isoprene degraders but amplified *isoA* genes from 15 characterized isoprene degraders, new isoprene-degrading isolates and from enrichment cultures set up with soil and leaf samples [42]. The *isoA* sequences retrieved from DNA obtained from environmental samples were compared with those from characterized isoprene degraders, including Gram-positive and Gram-negative strains, revealing that sequences were relatively highly conserved (>86% identity at the derived amino acid level). However, they could be broadly separated into two phylogenetic groups, those from the terrestrial environment and estuary head compared with those from marine environments, including the mouth of the Colne Estuary [42]. Genomic analysis of the increasing number and diversity of isoprene degraders being isolated (Murrell, Mejia-Flores, Murphy, McGenity et al. unpublished) will add validated sequences to the *isoA* database, and undoubtedly these *isoA* primer sets will require further refinement to avoid any potential cross-reactivity with other related soluble di-iron center containing oxygenases, but currently this approach shows great promise for detecting new isoprene degraders in many environments.

A powerful cultivation-independent technique to link the identity and function of microbes in the environment is DNA-stable isotope probing (DNA-SIP; [73, 74]). This approach has been used to identify active isoprene degraders in terrestrial and marine environments. Microcosms containing soil from the vicinity of Willow trees (*Salix fragilis*) were incubated with ^13^C-labeled isoprene. After enrichment with this “heavy” isoprene, DNA was extracted from soil and the ^13^C-labeled DNA arising from the incorporation of the target substrate into active isoprene degraders in this soil was separated by buoyant density ultracentrifugation. Analysis of 16S rRNA genes in DNA from unenriched soil revealed a typically diverse soil community, whereas the ^13^C-labeled DNA from isoprene-enriched soil was substantially enriched in 16S rRNA gene sequences from *Rhodococcus* species such as *R. wratislaviensis, R. koreensis*, and *R. globerulus* [42]. Sequences from members of the Betaproteobacteria, *Comamonas* spp. and *Variovorax* spp. were also enriched. Interestingly, further SIP experiments with cells washed from leaves of White Poplar (*Populus alba*) and shotgun metagenomics of the ^13^C-labeled DNA arising from incubations with ^13^C-isoprene have enabled the assembly and retrieval of a substantial portion of the genome of an isoprene-degrading *Variovorax* species that also contains the *iso* metabolic gene clusters *isoABCDEF* and *isoGHIJ* (Crombie et al. unpublished). The *isoABCDEF* gene cluster, recovered by DNA-SIP methods from this *Variovorax* sp., has also been shown to be a *bona-fide* isoprene monooxygenase since it has isoprene oxidation activity when expressed from a plasmid in a non-isoprene degrading *Rhodococcus* host. Analysis of metatranscriptome data from the same isoprene enrichments also confirmed the expression of these *iso* metabolic genes from *Variovorax* (Crombie et al. unpublished). These data suggested that *Variovorax*, a metabolically-versatile genus that is widespread in the terrestrial environment, could also be a major player in the isoprene cycle. Targeted cultivation has now enabled the isolation of an isoprene-degrading *Variovorax* from leaves, which provides a second (Gram-negative) model organism with which to analyse the regulation of isoprene metabolism (Murrell, McGenity, Crombie et al. unpublished).

The potential for isoprene degradation in marine and estuarine samples from the Colne Estuary, UK, has also been investigated using DNA-SIP. Surface sediments were incubated with ^13^C-labeled isoprene and consumption of isoprene was monitored by gas chromatography. Analysis of 16S rRNA genes amplified by PCR from ^13^C-DNA retrieved from these SIP enrichments at two time-points showed the development of communities dominated by Actinobacteria, including members of the genera *Mycobacterium, Rhodococcus, Microbacterium,* and *Gordonia* [61]. Particularly high enrichments of *Microbacterium* were noted in later time-points in SIP incubations. Enrichment and isolation studies with samples from the Colne Estuary yielded a number of isolates including *Gordonia* sp. i37 and *Mycobacterium* sp. AT1, representative of the DNA sequences retrieved in DNA-SIP experiments, which were capable of rapid and robust growth on isoprene [60, 61]. The genomes of both of these actinobacterial isolates contained *iso* gene clusters with the same gene arrangement and significant identity (55–87% at the amino acid level) to the corresponding genes from *Rhodococcus* strain AD45 (Fig. 4). In addition, a second SDIMO was identified elsewhere in the genomes of both *Gordonia* sp. i37 and *Mycobacterium* sp. AT1. Based on gene layout and sequence, the latter enzymes were predicted to belong to group V of the SDIMO family [62], most similar to propane monoxygenase (PrMO) from *Gordonia* TY5 ([75]; 98–100% amino acid identity) and phenol/propane monooxygenase from *Mycobacterium goodii* sp. 12523 ([76]; 88–97% identity), respectively. When tested, both strains also grew on propane. Expression studies showed that in both strains, isoprene oxidation is carried out by IsoMO and not PrMO, the ability to oxidize and grow on isoprene is an inducible trait and that isoprene- and propane-oxidizing ability is specific to cells grown on the corresponding substrate [61].

## Perspectives

Our understanding of the microbial methane cycle, represented by thousands of papers, is still producing surprises, including novel mechanisms for the production and consumption of methane. Despite isoprene being released into our biosphere and atmosphere in the same large amounts as methane and having major impacts on the climate, its microbial cycle is only starting to be understood. Addressing the fundamental and applied questions (as outlined in Box 1) will help *inter alia* to improve global models of isoprene flux, help us to learn how this may be affected by environmental change, uncover novel anabolic and catabolic pathways of environmental and biotechnological importance, and facilitate approaches to mitigate isoprene production.

Box 1 Key aspects of the microbial isoprene cycle that merit further exploration 1. Contribution of microbes to global isoprene production, interactions, and the elusive bacterial isoprene synthase

The enzyme(s) responsible for isoprene production in bacteria, and in any organism other than higher plants, remains to be discovered.

Given that soils are isoprene sinks and that many soil microbes produce isoprene, there is probably a cryptic isoprene cycle in terrestrial (and perhaps aquatic) environments, i.e., with substantial amounts of isoprene being consumed before it can escape to the atmosphere.

Isoprene may be a valuable source of carbon and energy for some microbes, and in the environment there may be interactions, i.e., close physical coupling, between producers and consumers so that the latter can capture this BVOC.

Isoprene elicits a behavioral response in some insects, but its signaling potential, especially involving microbes in aquatic environments, has received little attention.

2. Aerobic and anaerobic isoprene biodegradation and novel aerobic isoprene-degrading microbes

Even in the best characterized isoprene degrader, *Rhodococcus* strain AD45, the full details of catabolism need to be worked out, in particular the steps downstream of the metabolite GMBA and the flux of carbon into central metabolism (Fig. 3), promoters, transcriptional activators and the nature of the inducer of the isoprene monooxygenase gene cluster (Fig. 4).

Evidence is emerging that a number of Actinobacteria can use isoprene aerobically as a source of carbon and energy, and the role of other bacterial phyla is increasingly being recognized, especially Proteobacteria such as *Variovorax* and *Methylobacterium* spp. (Murrell, McGenity, Crombie, Murphy unpub.; [77]). It is important to establish the phylogenetic breadth of isoprene-degrading microbes, including a possible contribution from Fungi and Archaea, and to investigate the potentially novel metabolic pathways possessed by these microbes.

Several monoterpenes can be degraded anaerobically [78], so isoprene might be similarly degraded. However, most isoprene is produced by oxygenic phototrophs, suggesting that anaerobic biodegradation might be only a minor sink for isoprene.

3. Niche differentiation among isoprene-degrading bacteria: low-affinity versus high-affinity degraders

Methanotrophs have a range of affinities for methane, and those with high affinity, and thus capable of utilizing methane at atmospheric concentrations, have so far evaded cultivation. Isoprene concentrations decrease dramatically from source organism to the atmosphere, and so attempts should be made to isolate, or investigate by other means, microbes with the capacity to consume atmospheric concentrations of isoprene.

4. New cultivation-independent approaches to investigate isoprene-degrading microbes

The development of *isoA* primers and availability of ^13^C isoprene [42] has allowed the investigation of uncultivated isoprene degraders. As indicated above, exploration of the diversity of isoprene degraders will allow refinement of current PCR primers and development of new ones. DNA-SIP has been used and ongoing metagenomic and metatranscriptomic analyses of isoprene-degrading communities are providing important insights. There is scope to apply a wide range of approaches to investigate the interactions involved in the isoprene cycle and gain insight into the functioning of those isoprene degraders that prove difficult to culture, using techniques such as single-cell labeling and identification (e.g., via Raman-FISH and single-cell sorting and genomics).

5. Structure/function of IsoA and other possible mechanisms of isoprene biodegradation

Isoprene monooxygenase is a soluble di-iron center monooxygenase and it will be interesting to compare its structure and function, particularly in regard to substrate specificity, with related monooxygenases such as soluble methane monooxygenase and alkene monooxygenases, and also with dioxygenases which have been shown to co-oxidize isoprene [43].

Co-oxidation of isoprene by bacteria that contain related oxygenases, but not isoprene monooxygenase or enzymes necessary for growth on isoprene, might also be important in removing isoprene from the environment and the use of specific inhibitors to elucidate their role in the isoprene cycle also warrants investigation.

6. Biotechnological aspects of isoprene metabolism

In addition to the biosynthesis of isoprene as an important commodity chemical, as discussed in the text, there are potential applications of isoprene biodegradation; for example, isoprene-degrading communities are being investigated for their ability to scrub isoprene from the atmosphere in certain work environments [39, 79, 80]. When biomass crops like willow or palm oil are planted near urban areas that are sources of nitrogen oxides, their isoprene production can have a detrimental effect on air quality, e.g., by leading to the formation of ozone [4]. Therefore, the possibility of using phyllosphere-dwelling, isoprene-degrading microbes to mitigate isoprene escape to the atmosphere should be considered.

## References

[1] Guenther AB, Jiang X, Heald CL, Sakulyanontvittaya T, Duhl T, Emmons LK (2012). The model of emissions of gases and aerosols from nature version 2.1 (MEGAN2.1): an extended and updated framework for modeling biogenic emissions. Geosci Model Dev.

[2] Pacifico F, Harrison SP, Jones CD, Sitch S (2009). Isoprene emissions and climate. Atmos Environ.

[3] Collins WJ, Derwent RG, Johnson CE, Sanderson MG, Stevenson DS (2002). The oxidation of organic compounds in the troposphere and their global warming potentials. Clim Change.

[4] Ashworth K, Wild O, Hewitt C (2013). Impacts of biofuel cultivation on mortality and crop yields. Nat Clim Change.

[5] Carlton AG, Wiedinmyer C, Kroll JH (2009). A review of secondary organic aerosol (SOA) formation from isoprene. Atmos Chem Phys.

[6] Heal MR, Kumar P, Harrison RM (2012). Particles, air quality, policy and health. Chem Soc Rev.

[7] Rohmer M (1999). The discovery of a mevalonate-independent pathway for isoprenoid biosynthesis in bacteria, algae and higher plants. Nat Prod Rep.

[8] Bentlage B, Rogers TS, Bachvaroff TR, Delwiche CF (2016). Complex ancestries of isoprenoid synthesis in dinoflagellates. J Eukaryot Microbiol.

[9] Exton DA, Suggett DJ, McGenity TJ, Steinke M (2013). Chlorophyll-normalized isoprene production in laboratory cultures of marine microalgae and implications for global models. Limnol Oceanogr.

[10] Fall R, Copley SD (2000). Bacterial sources and sinks of isoprene, a reactive atmospheric hydrocarbon. Environ Microbiol.

[11] Kuzma J, Nemecek-Marshall M, Pollock WH, Fall R (1995). Bacteria produce the volatile hydrocarbon isoprene. Curr Microbiol.

[12] Bäck J, Aaltonen H, Hellén H, Kajos MK, Patokoski J, Taipale R (2010). Variable emissions of microbial volatile organic compounds (MVOCs) from root-associated fungi isolated from Scots pine. Atmosph Environ.

[13] Sharkey TD, Wiberley AE, Donohue AR (2008). Isoprene emission from plants: why and how. Ann Bot.

[14] Schöller C, Molin S, Wilkins K (1997). Volatile metabolites from some Gram-negative bacteria. Chemosphere.

[15] Schöller CEG, Gürtler H, Pederson R, Molin S, Wilkins K (2002). Volatile metabolites from actinomycetes. J Agric Food Chem.

[16] Sivy TL, Shirk MC, Fall R (2002). Isoprene synthase activity parallels fluctuations of isoprene release during growth of *Bacillus subtilis*. Biochem Biophys Res Commun.

[17] Wagner WP, Nemecek-Marshall M, Fall R (1999). Three distinct phases of isoprene formation during the growth and sporulation of *Bacillus subtilis*. J Bacteriol.

[18] Michelozzi M, Raschi A, Tognetti R, Tosi L (1997). Ecoethological analysis of the interaction between isoprene and the behavior of Collembola. Pedobiologia.

[19] Hess BM, Xue J, Markillie LM, Taylor RC, Wiley HS, Ahring BK (2013). Coregulation of terpenoid pathway genes and prediction of isoprene production in *Bacillus subtilis* using transcriptomics. PLoS One.

[20] Xue J, Ahring BK (2011). Enhancing isoprene production by genetic modification of the 1-deoxy-D-xylulose-5-phosphate pathway in *Bacillus subtilis*. Appl Environ Microbiol.

[21] Köksal M, Zimmer I, Schnitzler JP, Christianson DW (2010). Structure of isoprene synthase illuminates the chemical mechanism of teragram atmospheric carbon emission. J Mol Biol.

[22] Ge D, Xue Y, Ma Y (2016). Two unexpected promiscuous activities of the iron–sulfur protein IspH in production of isoprene and isoamylene. Microb Cell Fact.

[23] Whited GM, Feher FJ, Benko DA, Cervin MA, Chotani GK, McAuliffe JC (2010). Technology update: Development of a gas-phase bioprocess for isoprene-monomer production using metabolic pathway engineering. Ind Biotechnol.

[24] Ye L, Lv X, Yu H (2016). Engineering microbes for isoprene production. Metab Eng.

[25] Sharkey TD, Yeh S (2001). Isoprene emission from plants. Annu Rev Plant Physiol Plant Mol Biol.

[26] Vickers CE, Gershenzon J, Lerdau MT, Loreto F (2009). A unified mechanism of action for volatile isoprenoids in plant abiotic stress. Nat Chem Biol.

[27] Sharkey T, Monson RK (2017). Isoprene research—60 years later, the biology is still enigmatic. Plant Cell Environ.

[28] Velikova V, Sharkey TD, Loreto F (2012). Stabilization of thylakoid membranes in isoprene-emitting plants reduces formation of reactive oxygen species. Plant Sig Behav.

[29] Laothawornkitkul J, Paul ND, Vickers CE, Possell M, Taylor JE, Mullineaux PM (2008). Isoprene emissions influence herbivore feeding decisions. Plant Cell Environ.

[30] Jones AMP, Shukla MR, Sherif SM, Brown PB, Saxena PK (2016). Growth regulating properties of isoprene and isoprenoid based essential oils. Plant Cell Rep.

[31] Cleveland CC, Yavitt JB (1997). Consumption of atmospheric isoprene in soil. Geophys Res Lett.

[32] Cleveland CC, Yavitt JB (1998). Microbial consumption of atmospheric isoprene in a temperate forest soil. Appl Environ Microbiol.

[33] Pegoraro E, Abrell L, Van Haren J, Barron-Gafford G, Grieve KA, Malhi Y (2005). The effect of elevated atmospheric CO_2_ and drought on sources and sinks of isoprene in a temperate and tropical rainforest mesocosm. Glob Change Biol.

[34] Pegoraro E, Rey ANA, Abrell L, Haren J, Lin G (2006). Drought effect on isoprene production and consumption in Biosphere 2 tropical rainforest. Glob Change Biol.

[35] Gray CM, Helmig D, Fierer N (2015). Bacteria and fungi associated with isoprene consumption in soil. Elem Sci Anth.

[36] Ewers J, Freierschroder D, Knackmuss HJ (1990). Selection of trichloroethene (TCE) degrading bacteria that resist inactivation by TCE. Arch Microbiol.

[37] van Ginkel CG, Welten HGJ, de Bont JAM (1987). Oxidation of gaseous and volatile hydrocarbons by selected alkene-utilizing bacteria. Appl Environ Microbiol.

[38] van Ginkel CG, de Jong E, Tilanus JWR, de Bont JAM (1987). Microbial oxidation of isoprene, a biogenic foliage volatile and of 1,3-butadiene, an anthropogenic gas. FEMS Microbiol Lett.

[39] Srivastva N, Shukla AK, Singh RS, Upadhyay SN, Dubey SK (2015). Characterization of bacterial isolates from rubber dump site and their use in biodegradation of isoprene in batch and continuous bioreactors. Bioresour Technol.

[40] van Hylckama Vlieg JET, Kingma J, van den Wijngaard AJ, Janssen DB (1998). A glutathione *S*-transferase with activity towards *cis*-1,2-dichloroepoxyethane is involved in isoprene utilization by *Rhodococcus* sp. strain AD45. Appl Environ Microbiol.

[41] van Hylckama Vlieg JET, Leemhuis H, Spelberg JHL, Janssen DB (2000). Characterization of the gene cluster involved in isoprene metabolism in *Rhodococcus* sp. strain AD45. J Bacteriol.

[42] El Khawand M, Crombie AT, Johnston A, Vavlline DV, McAuliffe JC, Latone JA (2016). Isolation of isoprene degrading bacteria from soils, development of *isoA* gene probes and identification of the active isoprene degrading soil community using DNA-stable isotope probing. Environ Microbiol.

[43] Boyd DR, Clarke D, Cleij MC, Hamilton JTG, Sheldrake GN (2000). Bacterial biotransformation of isoprene and related dienes. Mon Chem.

[44] Exton DA, Suggett DJ, Steinke M, McGenity TJ (2012). Spatial and temporal variability of biogenic isoprene emissions from a temperate estuary. Glob Biogeochem Cycl.

[45] Moore RM, Wang L (2006). The influence of iron fertilization on the fluxes of methyl halides and isoprene from ocean to atmosphere in the SERIES experiment. Deep-Sea Res Pt II.

[46] Shaw SL, Gantt B, Meskhidze N (2010). Production and emissions of marine isoprene and monoterpenes: a review. Adv Meteorol.

[47] Hackenberg SC, Andrews SJ, Airs R, Arnold SR, Bouman HA, Brewin RJW (2017). Potential controls of isoprene in the surface ocean. Glob Biogeochem Cycles.

[48] Arnold SR, Spracklen DV, Williams J, Yassaa N, Sciare J, Bonsang B (2009). Evaluation of the global oceanic isoprene source and its impacts on marine organic carbon aerosol. Atmos Chem Phys.

[49] Luo G, Yu F (2010). A numerical evaluation of global oceanic emissions of α-pinene and isoprene. Atmos Chem Phys.

[50] Palmer PI, Shaw SL (2005). Quantifying global marine isoprene fluxes using MODIS chlorophyll observations. Geophys Res Lett.

[51] Gantt B, Meskhidze N, Kamykowski D (2009). A new physically-based quantification of marine isoprene and primary organic aerosol emissions. Atmos Chem Phys.

[52] Meskhidze N, Sabolis A, Reed R, Kamykowski D (2015). Quantifying environmental stress-induced emissions of algal isoprene and monoterpenes using laboratory measurements. Biogeosci.

[53] Ciuraru R, Fine L, Pinxteren M, D'Anna B, Herrmann H, George C (2015). Unravelling new processes at interfaces: photochemical isoprene production at the sea surface. Environ Sci Technol.

[54] Exton DA, McGenity TJ, Steinke M, Smith DJ, Suggett DJ (2015). Uncovering the volatile nature of tropical coastal marine ecosystems in a changing world. Glob Change Biol.

[55] Ooki A, Nomura D, Nishino S, Kikuchi T, Yokouchi Y (2015). A global-scale map of isoprene and volatile organic iodine in surface seawater of the Arctic, Northwest Pacific, Indian, and Southern Oceans. J Geophys Res Oceans.

[56] Booge D, Marandino C, Schlundt C, Palmer PI, Schlundt M, Atlas EL (2016). Can simple models predict large-scale surface ocean isoprene concentrations?. Atmos Chem Phys.

[57] Srikanta Dani KG, Silva Benavides AM, Michelozzi M, Peluso G, Torzillo G, Loreto F (2017). Relationship between isoprene emission and photosynthesis in diatoms, and its implications for global marine isoprene estimates. Mar Chem.

[58] Steinke M, Hodapp B, Subhan R, Bell TG, Martin-Creuzburg D (2017). Oligotrophic lake constance is a source of the biogenic volatile organic compounds isoprene and dimethyl sulfide. Sci Rep.

[59] Srikanta Dani KG, Loreto F (2017). Trade-off between dimethyl sulfide and isoprene emissions from marine phytoplankton. Trends Plant Sci.

[60] Acuña Alvarez L, Exton DA, Suggett DJ, Timmis KN, McGenity TJ (2009). Characterization of marine isoprene-degrading communities. Environ Microbiol.

[61] Johnston A, Crombie AT, El Khawand M, Sims L, Whited GM, McGenity TJ (2017). Identification and characterisation of isoprene-degrading bacteria in an estuarine environment. Environ Microbiol.

[62] Holmes AJ, Coleman NV (2008). Evolutionary ecology and multidisciplinary approaches to prospecting for monooxygenases as biocatalysts. Antonie Van Leeuwenhoek.

[63] Leahy JG, Batchelor PJ, Morcomb SM (2003). Evolution of the soluble diiron monooxygenases. FEMS Microbiol Rev.

[64] van Hylckama Vlieg JET, Kingma J, Kruizinga W, Janssen DB (1999). Purification of a glutathione *S*-transferase and a glutathione conjugate-specific dehydrogenase involved in isoprene metabolism in *Rhodococcus* sp. strain AD45. J Bacteriol.

[65] Shennan JL (2006). Utilisation of C_2_–C_4_ gaseous hydrocarbons and isoprene by microorganisms. J Chem Technol Biotechnol.

[66] Mattes TE, Alexander AK, Coleman NV (2010). Aerobic biodegradation of the chloroethenes: pathways, enzymes, ecology, and evolution. FEMS Microbiol Rev.

[67] Kottegoda S, Waligora E, Hyman M (2015). Metabolism of 2-methylpropene (isobutylene) by the aerobic bacterium *Mycobacterium* sp. strain ELW1. Appl Environ Microbiol.

[68] Crombie AT, El Khawand M, Rhodius VA, Fengler KA, Miller MC, Whited GM (2015). Regulation of plasmid-encoded isoprene metabolism in *Rhodococcus*, a representative of an important link in the global isoprene cycle. Environ Microbiol.

[69] Chen Y, Ding Y, Yang L, Yu J, Liu G, Wang X (2014). Integrated omics study delineates the dynamics of lipid droplets in *Rhodococcus opacus* PD630. Nucleic Acids Res.

[70] Harwood CS, Parales RE (1996). The β-ketoadipate pathway and the biology of self-identity. Annu Rev Microbiol.

[71] Murphy G. Isoprene degradation in the terrestrial environment. PhD Thesis, University of Essex; 2017.

[72] McDonald IR, Bodrossy L, Chen Y, Murrell JC (2008). Molecular ecology techniques for the study of aerobic methanotrophs. Appl Environ Microbiol.

[73] Radajewski S, Ineson P, Parekh NR, Murrell JC (2000). Stable-isotope probing as a tool in microbial ecology. Nature.

[74] Dumont M, Murrell JC (2005). Stable isotope probing—linking microbial identity to function. Nat Rev Microbiol.

[75] Kotani T, Yamamoto T, Yurimoto H, Sakai Y, Kato N (2003). Propane monooxygenase and NAD^+^-dependent secondary alcohol dehydrogenase in propane metabolism by *Gordonia* sp. strain TY-5. J Bacteriol.

[76] Furuya T, Hirose S, Osanai H, Semba H, Kino K (2011). Identification of the monooxygenase gene clusters responsible for the regioselective oxidation of phenol to hydroquinone in mycobacteria. Appl Environ Microbiol.

[77] Srivastva N, Vishwakarma P, Bhardwaj Y, Singh A, Manjunath K, Dubey SK (2017). Kinetic and molecular analyses reveal isoprene degradation potential of *Methylobacterium* sp. Bioresour Technol.

[78] Marmulla R, Harder J (2014). Microbial monoterpene transformations—a review. Front Microbiol.

[79] Srivastva N, Singh RS, Upadhyay SN, Dubey SK (2016). Degradation kinetics and metabolites in continuous biodegradation of isoprene. Bioresour Technol.

[80] Srivastva N, Singh RS, Dubey SK (2017). Efficacy of wood charcoal and its modified form as packing media for biofiltration of isoprene. J Environ Manage.

[81] Vinokur JM, Korman TP, Cao Z, Bowie JU (2014). Evidence of a novel mevalonate pathway in archaea. Biochemistry.

[82] Lange BM, Rujan T, Martin W, Croteau R (2000). Isoprenoid biosynthesis: the evolution of two ancient and distinct pathways across genomes. Proc Natl Acad Sci USA.

[83] Steinke M, Exton DA, McGenity TJ (2011). Challenges to the bio(geo)chemist: marine gases. Biochemist.

[84] Wyche KP, Ryan RC, Hewitt CN, Alfarra MR, Carr T, Hewitt CN (2014). Emissions of biogenic volatile organic compounds and subsequent photochemical production of secondary organic aerosol in mesocosm studies of temperate and tropical plant species. Atmos Chem Phys.

